# Screening and Whole-Genome Sequencing of Two* Streptomyces* Species from the Rhizosphere Soil of Peony Reveal Their Characteristics as Plant Growth-Promoting Rhizobacteria

**DOI:** 10.1155/2018/2419686

**Published:** 2018-09-05

**Authors:** Chengqiang Wang, Yun Wang, Jinjin Ma, Qihui Hou, Kai Liu, Yanqin Ding, Binghai Du

**Affiliations:** College of Life Sciences and Shandong Key Laboratory of Agricultural Microbiology and National Engineering Laboratory for Efficient Utilization of Soil and Fertilizer Resources, Shandong Agricultural University, Tai'an, China

## Abstract

Two bacteria,* Streptomyces albireticuli* MDJK11 and* S. alboflavus* MDJK44, which are potential plant growth-promoting rhizobacteria against pathogenic fungi were isolated from the rhizosphere soil of peony in Shandong, China. Their biological characteristics and complete genome sequences were reported in this study. The total genome size of MDJK11 was only 8.14 Mb with 6,550 protein-coding genes and a high GC content of 72.8 mol%. The MDJK44 genome comprises a 9.62 Mb chromosome with 72.1 mol% GC content, 7,285 protein-coding genes, and two plasmids. Some gene sequences in these two genomes were analyzed to be heterologously obtained by horizontal transfer. Gene or gene cluster candidates responding to secondary metabolites production, antimicrobial activities, and plant growth-promoting capacities were also analyzed in this paper. The genomic information and biological characteristics will facilitate the understanding and application of* S. albireticuli* and* S. alboflavus* species as biocontrol agents in future agriculture.

## 1. Introduction

Nowadays, the yield and quality of many ornamental plants, pharmaceutical plants, vegetables, fruits, and crops are decreased because of plant diseases caused by soil-borne pathogens [1–4]. Furthermore, a variety of chemical pesticides and fertilizers have been chronically used for farming, thus causing the quality reduction of agricultural products [5], pathogen resistance to chemicals [1], and environmental pollution[6]. Peony (*Paeonia suffruticosa*) is a national flower of China, which has important ornamental, economic, and medicinal value. The flowers and roots of peony can be used as herbal medicine because of the contained components, such as flavonoids, phenolic components, and microelements [7]. They can resist oxidative damage, enhance skin flexibility, regulate menstruation and dysmenorrheal for women, and so on [7, 8]. Due to long-term cultivation and unsuitable management, peony has been obviously affected by soil-borne diseases, which led to output reduction [9, 10].

Plant growth-promoting rhizobacteria (PGPR) are a group of bacteria which localize in the plant rhizosphere and play important roles in controlling soil-borne diseases, promoting plant growth, increasing crop yield, improving the soil environment, and so on [11–14]. As a kind of gram-positive actinomycetes,* Streptomyces *species are abundant in the soil, many of which are important members of PGPR and some species of them have been used in agriculture as biocontrol agents [15]. Some* Streptomyces *strains could promote plants to acquire nutrients [16, 17] and also directly produce many active compounds for plants, including antibiotics, volatile organic compounds, and hydrolytic enzymes [18, 19]. In recent years, PGPR such as* Streptomyces *species have attracted much attention for biological control of soil-borne pathogens [20, 21].* Streptomyces alboflavus* has been shown to inhibit some pathogenic fungi and gram-positive bacteria by a few published studies.* S. alboflavus *TD-1 was verified to produce volatile organic compounds to inhibit* Fusarium moniliforme* Sheldon,* Aspergillus flavus*,* Penicillium citrinum*, and so on [22, 23].* S. alboflavus *313 has been shown to produce cyclic hexapeptides to inhibit some gram-positive bacteria [24, 25]. However, up to now, the biocontrol capacities and mechanisms of* S. alboflavus* as PGPR have not been effectively investigated. For species* S. albireticuli*, its biological research is even less to date [26] and its biological control capacity and genetic mechanism as PGPR have not yet been exploited.

In this study, strains MDJK11 and MDJK44 were isolated from the rhizosphere soil of peony in Shandong, China, which showed inhibition activity against pathogenic fungi* F. solani*. These two strains were analyzed based on physiological and biochemical properties and 16S rRNA gene sequence analysis. To further identify these strains and study their genetic basis for biocontrol ability, we carried out the complete genome sequencing and then investigated potential gene clusters for producing antimicrobial metabolites.

## 2. Materials and Methods

### 2.1. Strain Isolation and Cultivation

The fresh soil of peony rhizosphere was serially diluted by sterilized water and then widely spread on the gauze No. 1 (GA) [27] medium with 3% potassium dichromate, and then the plates were incubated at 28°C for 4-7 days. The dominant colonies were isolated and then triply streaked to obtain the absolute single colonies [28].* F. solani*, which is the pathogen of peony root rot was cultivated using potato dextrose agar (PDA) [27] medium at 28°C.

### 2.2. The Antifungal Experiments

The activities of strains MDJK11 and MDJK44 against* F. solani* were verified as follows [29]: Fresh* F. solani* with the size of 6 mm in diameter was inoculated in the center of a PDA agar plate and then cultivated at 28°C to obtain a strain lawn with the size of 1 cm or less in diameter. Then, strain MDJK11 or MDJK44 was inoculated near one side of* F. solani* at a distance of 1 cm. After incubation at 28°C for another 3 to 7 days, the inhibition zones were observed.

### 2.3. Test of Physiological and Biochemical Properties

Parts of physiological and biochemical characteristics of strains MDJK11 and MDJK44 were determined. For instance, the tests of gram stain, starch hydrolysis, gelatin liquefaction, and hydrogen sulfide production were performed as formal processes reported [30]. Catalase activity was determined by assessing the production of bubbles after adding 3% H_2_O_2_ [31]. Nitrate reduction, indole production, and carbon utilization were tested using the bacteria microbiochemical identification tubes (HOPEBIO, China) [31].

### 2.4. Construction of Phylogenetic Tree

The genomic DNA of strains MDJK11 and MDJK44 was extracted using the method of Tris-EDTA (TE) boiling [32]. Polymerase chain reaction (PCR) was performed to amplify 16S rRNA sequences using primers 27F (5′-AGAGTTTGATCCTGGCTCAG-3) and 1492R (5′-GGTTACCTTGTTACGACTT-3′). The identified sequences were then aligned to the National Center for Biotechnology Information (NCBI) (http://www.ncbi.nlm.nih.gov). A neighbor-joining phylogenetic tree was constructed by MEGA 5.0 [33] with some members of genus* Streptomyces* based on 16S rRNA gene sequences.

### 2.5. Genome Sequencing and Analyzing

The complete genomes of strains MDJK11 and MDJK44 were sequenced by the third generation sequencing technology based on the PacBio platform. The whole-genome sequences were assembled by software HGAP. The average nucleotide identity (ANI) analysis was performed by JSpecies1.2.1 [34]. The NCBI Prokaryotic Genomes Automatic Annotation Pipeline (PGAAP) was used to perform the gene annotation. The gene functions were further analyzed by BLASTP using five databases: Cluster of Orthologous Groups (COG) of proteins, Gene Ontology (GO), Kyoto Encyclopedia of Genes and Genomes (KEGG), Nonredundant Protein Database (NR), and Swiss-Prot. The genomic islands (GIs) were predicted by IslandViewer 4 which integrates four different genomic island prediction methods: IslandPick, IslandPath-DIMOB, SIGI-HMM, and Islander [35]. The potential secondary metabolite biosynthetic gene clusters were predicted out using antiSMASH v.4.1.0 [36]. The qualitative analysis of these two strains for producing siderophores was performed on CAS-agar plates, according to the methods described in [14].

## 3. Results

### 3.1. Identification of Two Actinomycetes MDJK11 and MDJK44

**Figure 1:**
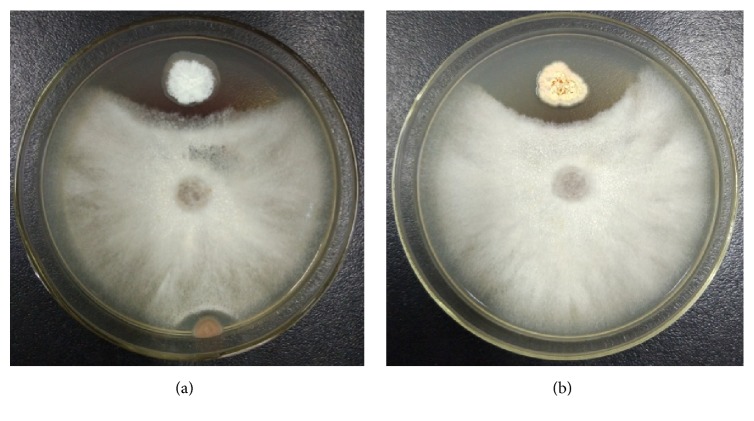
In vitro antifungal activities of strains MDJK11 (a) and MDJK44 (b) against* F. solani*. Newly cultivated hyphal plugs of* F. solani* were placed on the center of PDA plates and incubated to obtain a 1 cm strain lawn in diameter at 28°C. Then, strains MDJK11 and MDJK44 were inoculated onto the top side of the plug at a distance of 1 cm and incubated for another 5 days.

**Figure 2:**
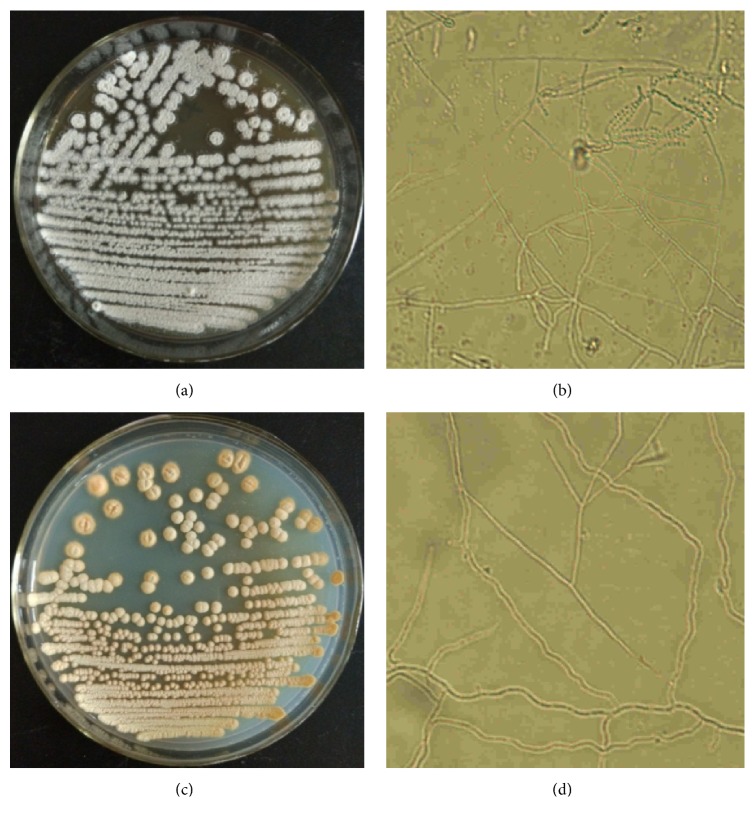
Morphological characteristics of strains MDJK11 and MDJK44. Colony morphologies of MDJK11 (a) and MDJK44 (c), and hypha morphologies (magnification 10 × 100) of MDJK11 (b) and MDJK44 (d) after incubation on GA medium for 14 d.

The strains were isolated from the rhizosphere soil of peony and then tested for biocontrol activities against the pathogen of peony root rot:* F. solani*. Only two strains named MDJK11 and MDJK44 showed robust inhibition on the growth of* F. solani* (Figure 1). Moreover, the biocontrol activity of MDJK44 was more stronger than that of MDJK11. Their morphology of colony and hypha were shown in Figure 2. Specifically, MDJK11 formed a dry, white-gray, and suborbicular colony. MDJK44 formed a dry, orange, and suborbicular colony. Both of them had obvious hyphae structure. Their morphologies of the colony and hypha belonged to typical actinomycetes.

Some physiological and biochemical properties of strains MDJK11 and MDJK44 were also studied (Table 1). They were all gram-positive strains. The catalase activity, citrate utilization, nitrate reduction, and sorbitol utilization were all positive for the two strains. They did not possess the capacity of gelatin liquefaction and hydrogen sulfide production. Some differences also existed between them. The strain MDJK44 could utilize inositol and propionate, but the strain MDJK11 did not.

**Figure 3:**
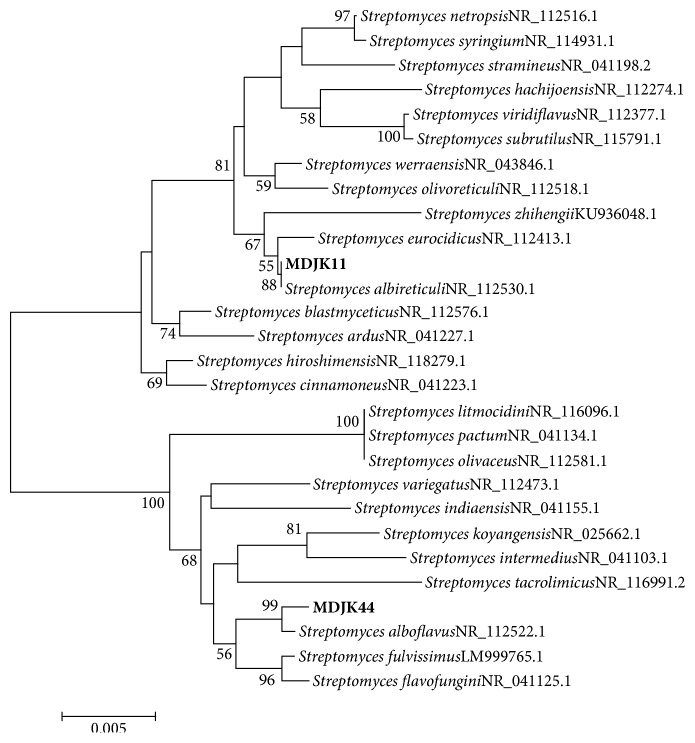
Neighbor-joining phylogenetic tree of strains MDJK11 and MDJK44 based on 16S rRNA gene sequences. The phylogenetic tree was constructed using the MEGA 5.0 program and evolutionary distances were computed by the Maximum Likelihood method. Bootstrap values (expressed as percentages of 1000 replications) >50% are indicated at the branch points. The scale bar indicates 0.005 nucleotide substitutions per site.

The phylogenetic analysis of strains MDJK11 and MDJK44 based on 16S rRNA sequences was conducted by MEGA 5.0 with related species (Figure 3) to show their phylogenetic relationships. These two strains were successfully clustered to genus* Streptomyces*. Moreover, the closest relatives of strains MDJK11 and MDJK44 were* S. albireticuli *NR_112530.1 and* S. alboflavus *NR_112522.1, respectively. These two strains could be identified to be species of* Streptomyces*.

### 3.2. General Genome Features of Strains MDJK11 and MDJK44

The complete genomes of strains MDJK11 and MDJK44 were sequenced by the third generation sequencing technology: PacBio platforms. The genome coverages of strains MDJK11 and MDJK44 were 88.0x and 102.0x, respectively. The software HGAP was used to assemble the whole-genome sequences. The genome of strain MDJK11 contained an 8.14 Mb chromosome with a GC content of 72.8 mol%, including 6550 protein-coding genes, 21 tRNA, 74 rRNA, and 3 other RNA (Table 2). No plasmid was found. Strain MDJK44 contained a 9.62 Mb chromosome with GC content of 72.1 mol%, including 7285 protein-coding genes, 18 tRNA, 67 rRNA, and 3 other RNA. Two native plasmids, pSJK1 and pSJK2, also existed (Table 2) and their GC contents were different from that of the chromosome. As shown in Supplementary S1, strains MDJK11 and MDJK44 resulted in higher ANI values with species* S. albireticuli* and* S. alboflavus*, respectively, which was comparable with the cut-off value 95% to distinguish different species. According to the genomic information, the two bacteria MDJK11 and MDJK44 were finally identified to be* S. albireticuli* and* S. alboflavus*, respectively.

**Figure 4:**
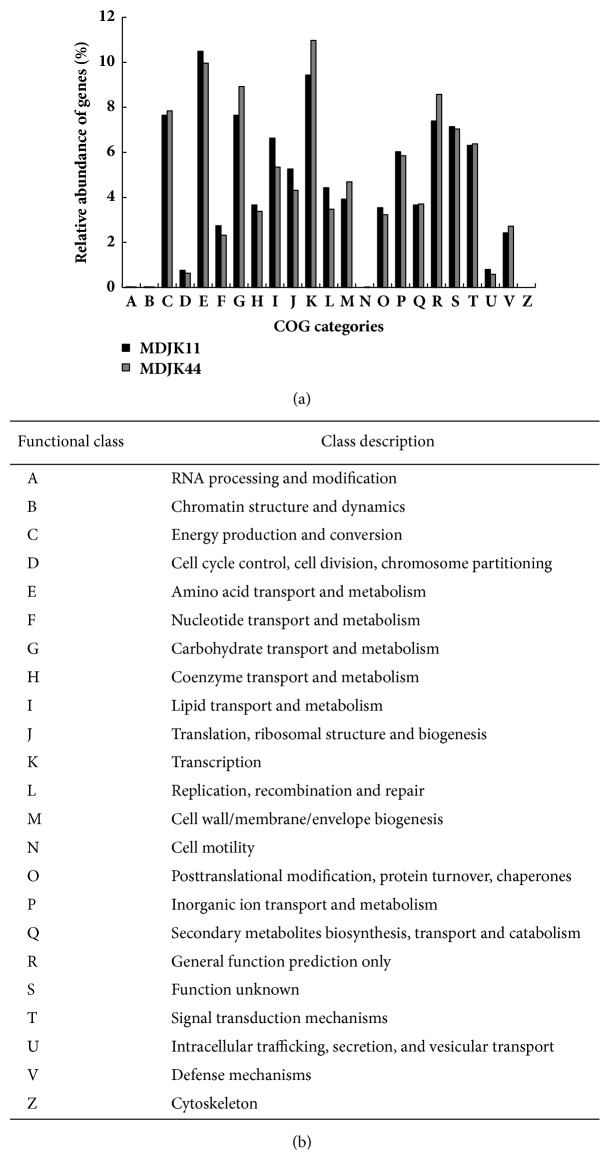
COG database annotation of strains MDJK11 and MDJK44. (a) The relative abundance of genes (%) in the two genomes. (b) COG functional classed.

There were total 3137 and 3966 genes that were assigned to the COG databases for strains MDJK11 and MDJK44, respectively (Figure 4). For these two strains, about half of genes were annotated by COG and the trend in different functional classes was similar. The genes encoding energy production and conversion, amino acid transport and metabolism, carbohydrate transport and metabolism, and transcription accounted for a large proportion (each more than 7%). The genes encoding amino acid transport and metabolism accounted for the largest proportion (10.49%) for strain MDJK11. The genes encoding transcription accounted for the largest proportion (10.97%) for strain MDJK44. This indicated the better absorption capacity and response ability of the two species for amino acids and carbohydrates in the living soil environment.

GIs could be related to a variety of functions, such as the symbiotic relationship, pathogenesis, and the biological adaptability [37]. Some GIs were found in the analyzed two genomes and their general information was listed in Table 3. The GIs were abundant in strain MDJK11 which had 21 GIs with the average length of 12737 bp. However, strain MDJK44 only contained 6 GIs, but the average length was up to 27530 bp. A part of the CDS sequences were predicted out in the GIs of the two strains, although many coded proteins were just described as hypothetical proteins. The majority of defined CDS in GIs of MDJK11 and MDJK44 were related to DNA operating enzymes and transcriptional or response regulators. In the GIs of MDJK11, a pair of excisionase and integrase with two transposases were found, which indicated the existence of horizontal transfer of genes. Two polyketide synthases were also found, which indicated the accumulation of external resistance. To compare GIs with that of MDJK11, MDJK44 contained six transposases and two transfer elements, which indicated more frequent native gene transfer. Interestingly, a series of urease synthetic genes were found in MDJK44. Furthermore, both strains possessed more than one phage tail protein, which indicated their ever target activities by phages.

### 3.3. Genetic Basis for Producing Antimicrobial and Plant Growth-Promoting Metabolites

Strains MDJK11 and MDJK44 were selected from the soil habitat due to their activities against pathogenic fungi (Figure 1), which indicated the existence of some important antimicrobial gene clusters. We also verified that these two strains could produce siderophores to chelate iron on CAS-ager plates (data shown in Supplementary S2), which indicated the existence of some siderophores production gene clusters. Many different types of secondary metabolite clusters were found in the two strains according to antiSMASH (v.4.1.0) (Tables 4 and 5). These clusters were mainly responsible for terpenoid synthesis and biological resistance. Moreover, some clusters were related to the types of nonribosomal peptide synthase (Nrps) and polyketide synthase (Pks). In the MDJK11 genome, a total of 37 secondary metabolic gene clusters were predicted, among which 30 gene clusters presented known function as similar gene clusters. The similarities of three gene clusters with ever reported clusters were 100%. Cluster 1 was responsible for 2-methylisoborneol (MIB) biosynthesis, cluster 13 was responsible for teleocidin B biosynthesis, and cluster 23 was responsible for ectoine biosynthesis. Both MIB and teleocidin B were important terpenoids and they were synthetized by terpene synthases[35, 38]. Ectoine was an effective microbial osmostress protectant, which could also serve as a versatile nutrient[39]. Furthermore, the similarities of cluster 7, cluster 10, cluster 22, cluster 30, cluster 31, and cluster 34 with their similar and known gene clusters were all more than 60%, which might indicate the biosynthesis ability of hopene, lantipeptide, cyclooctatin (a kind of siderophore), albachelin, and candicidin. More important, candicidin was an aromatic heptane, which was currently identified and exhibited a robust antifungal activity [40]. In the MDJK44 genome, a total of 29 secondary metabolic gene clusters were figured out and the function of 24 gene clusters could be predicted. The similarities of cluster 7 and cluster 13 with ever reported clusters were 100%. They were responsible for ectoine and albaflavenone (a novel antibiotic sesquiterpene) [41] biosynthesis, respectively. All of the predicted similarities of cluster 2, cluster 8, cluster 18, cluster 19, cluster 20, cluster 23, and cluster 25 with their similar and ever reported gene clusters were more than 60%, which might indicate the biosynthesis ability of pamamycin, desferrioxamine B, hopene, marineosin, neoaureothin, coelichelin, and griseobactin. The biosynthesis genes of three kinds of siderophores desferrioxamine B, coelichelin, and griseobactin in strain MDJK44 indicated its ability to improve the absorption of irons for promoting plant growth [14, 42]. Pamamycins, marineosin, and neoaureothin might be responsible for the antimicrobial activities. However, no effective secondary metabolic gene clusters were identified in the two plasmids of strain MDJK44. Our findings highlighted the molecular genetic mechanism of* S. albireticuli* and* S. alboflavus* strains for biocontrol ability.

The genomes of strains MDJK11 and MDJK44 also harbored other plant growth-promoting genes and could produce some beneficial substances, including glucosidase, phytase, and phosphatase. The genes that were likely involved in molecular communication, degradation of harmful substances, and environmental responses were also found.

### 3.4. Nucleotide Sequence Accession Numbers

The complete genomic sequence of* S. albireticuli* MDJK11 has been deposited in GenBank under accession number CP021744. The complete genomic sequences of* S. alboflavus* MDJK44 have been deposited in GenBank under accession numbers CP021748, CP023976, and CP023977.

## 4. Discussion

Peony is the national flower of China, which contains not only important ornamental effect, but also important economic and medicinal value. In recent years, due to long-term cultivation and continuous cropping, peony was increasingly affected by soil-borne diseases. Especially, the spread of root rot seriously affected the yield and quality of peony. In this study, we successfully screened and identified two actinomycete species,* S. albireticuli *MDJK11 and* S. alboflavus *MDJK44, from the rhizosphere soil of peony.

Biocontrol experiments indicated that MDJK11 and MDJK44 can effectively inhibit the pathogen of peony root rot* F. solani*. Morphological observation and phylogenetic analysis showed that the two strains were related to genus* Streptomyces*. To exactly recognize and understand the two species, we obtained their complete genome sequences and calculated their ANI values with related species. This is the first time to identify the complete genome sequences for the two species* S. albireticuli* and* S. alboflavus*. According to the genomic information, genes for primary and secondary metabolism were then annotated. Among them, a variety of horizontally transferred genes were identified, which predicted the long time coevolution in soil habitat with other species. More remarkable, the genetic basis of these two species as PGPR for producing antimicrobial and plant growth-promoting metabolites was also predicted. The production of siderophores of these two strains meant their capacity to improve the absorption of iron by plants and also repress the phytopathogens [14]. It is noteworthy that many kinds of terpene synthases are widely distributed in these two strains to have many potential values [41]. Some gene clusters in these two strains might improve the resistance of peony for the environmental stress. For example, the presence of synthetic gene cluster of ectoine [39] in both strains might improve the salt stress of peony in saline-alkali land. At present, the research on* Streptomyces* strains as PGPR has been carried out [16, 17], but there were relatively little biocontrol study and genome research about species* S. albireticuli *and* S. alboflavus.* The strains MDJK11 and MDJK44 just provide the biocontrol and growth-promoting capacities of species* S. albireticuli *and* S. alboflavus*, respectively. The complete genome sequences of strains MDJK11 and MDJK44 also revealed the entirely genetic basis of the two species as new biocontrol agents. We further provide the possibility of* Streptomyces* resource for studying and producing new microbial fertilizers, which might have good prospect in agricultural activities.

## 5. Conclusions

In this study, two actinomycete species* S. albireticuli* MDJK11 and* S. alboflavus* MDJK44 were isolated and identified, which has effective function for antagonizing the root rot pathogen* F. solani* of peony. The complete genome sequences of them were obtained, were analyzed, and also presented the genetic basis for the biofunction of species* S. albireticuli* and* S. alboflavus* as PGPR.

## Figures and Tables

**Table 1:** Physiological and biochemical traits of MDJK11 and MDJK44.

characteristic	MDJK11	MDJK44
Gram stain	**+**	**+**
Starch hydrolysis	**+**	**+**
Gelatin liquefaction	−	−
Propionate utilization	−	**+**
Citrate utilization	**+**	**+**
Indole production	−	−
Catalase activity	**+**	**+**
Hydrogen sulfide production	−	−
Nitrate reduction	**+**	**+**
Inositol utilization	−	**+**
Sorbitol utilization	**+**	**+**

**Table 2:** The general genome feature of strains MDJK11 and MDJK44.

Feature	Value			
	MDJK11	MDJK44	pSJK1	pSJK2
Genome size (Mb)	8.14	9.62	0.26	0.09
GC content (%)	72.8	72.1	70.1	67.5
Total number of genes	6931	8379	284	81
Protein-coding genes	6550	7285	284	81
rRNA number	21	18	0	0
tRNA number	74	67	0	0
Other RNA number	3	3	0	0
Pseudo Genes (total)	283	1006	0	0

**Table 3:** Genomic islands in strains MDJK11 and MDJK44.

Strain	GIs number	GIs total length (bp)	Average length (bp)
MDJK11	21	267,481	12,737
MDJK44	6	165,181	27,530

**Table 4:** The potential gene clusters encoding the secondary metabolites in strain MDJK11.

Cluster	Type	Most similar known cluster	Gene similarity (%)
Cluster 1	Terpene	2-methylisoborneol_biosynthetic_gene_cluster	100
Cluster 2	Siderophore-T1pks-Lasso peptide-Nrps	Chlorothricin_biosynthetic_gene_cluster	16
Cluster 3	Nrps	-	
Cluster 4	Other	Paromomycin_biosynthetic_gene_cluster	15
Cluster 5	Bacteriocin	-	
Cluster 6	T2pks	Iso*ﬂ*urano naphthoquinone_biosynthetic_gene_cluster	44
Cluster 7	Terpene	2-methylisoborneol_biosynthetic_gene_cluster	75
Cluster 8	T1pks-Nrps	Griseobactin_biosynthetic_gene_cluster	47
Cluster 9	Lantipeptide	Cetoniacytone_A_biosynthetic_gene_cluster	19
Cluster 10	Terpene	Hopene_biosynthetic_gene_cluster	76
Cluster 11	T2pks	Granaticin_biosynthetic_gene_cluster	32
Cluster 12	Nrps	Kanamycin_biosynthetic_gene_cluster	6
Cluster 13	Indole-Nrps	Teleocidin_B_biosynthetic_gene_cluster	100
Cluster 14	Other	JBIR-34,_JBIR-35_biosynthetic_gene_cluster	8
Cluster 15	T1pks-Nrps	Midecamycin_biosynthetic_gene_cluster	41
Cluster 16	Siderophore	-	
Cluster 17	Melanin	Melanin_biosynthetic_gene_cluster	28
Cluster 18	T3pks	Herboxidiene_biosynthetic_gene_cluster	2
Cluster 19	Butyrolactone-Otherks	Thienodolin_biosynthetic_gene_cluster	14
Cluster 20	T1pks	Saprolmycin_biosynthetic_gene_cluster	8
Cluster 21	Melanin	Melanin_biosynthetic_gene_cluster	28
Cluster 22	Lantipeptide-Terpene	SapB_biosynthetic_gene_cluster	75
Cluster 23	Ectoine	Ectoine_biosynthetic_gene_cluster	100
Cluster 24	Siderophore	-	
Cluster 25	Other	A-503083_biosynthetic_gene_cluster	7
Cluster 26	T1pks-Nrps	-	
Cluster 27	Thiopeptide	-	
Cluster 28	Aryl polyene	Pyrrolomycin_biosynthetic_gene_cluster	18
Cluster 29	T2pks-T1pks-Transatpks-Butyrolactone-Nrps	Rabelomycin_biosynthetic_gene_cluster	35
Cluster 30	T1pks-Terpene-Siderophore	Cyclooctatin_biosynthetic_gene_cluster	75
Cluster 31	Nrps	Albachelin_biosynthetic_gene_cluster	70
Cluster 32	Terpene-T3pks-Lantipeptide-T1pks	Pactamycin_biosynthetic_gene_cluster	5
Cluster 33	Terpene	-	
Cluster 34	T1pks-Aryl polyene-Nrps	Candicidin_biosynthetic_gene_cluster	66
Cluster 35	Nrps	Azinomycin_B_biosynthetic_gene_cluster	23
Cluster 36	Bacteriocin-T1pks-Ectoine	Chloramphenicol_biosynthetic_gene_cluster	23
Cluster 37	T1pks-Terpene-Nrps	Xiamycin_biosynthetic_gene_cluster	20

“-” corresponds to no similar known clusters.

**Table 5:** The potential gene clusters encoding the secondary metabolites in strain MDJK44.

Cluster	Type	Most similar known cluster	Gene similarity (%)
Cluster 1	T1pks	-	
Cluster 2	T2pks	Pamamycin_biosynthetic_gene_cluster	95
Cluster 3	T1pks	Elaiophylin_biosynthetic_gene_cluster	25
Cluster 4	Terpene	-	
Cluster 5	T3pks	Akaeolide_biosynthetic_gene_cluster	12
Cluster 6	Nrps	Steffimycin_biosynthetic_gene_cluster	25
Cluster 7	Ectoine	Ectoine_biosynthetic_gene_cluster	100
Cluster 8	Siderophore	Desferrioxamine_B_biosynthetic_gene_cluster	83
Cluster 9	Melanin	Melanin_biosynthetic_gene_cluster	28
Cluster 10	Butyrolactone-Otherks	Pactamycin_biosynthetic_gene_cluster	11
Cluster 11	Melanin	Istamycin_biosynthetic_gene_cluster	5
Cluster 12	Phosphonate-Butyrolactone-Nrps	Neocarzinostatin_biosynthetic_gene_cluster	8
Cluster 13	Terpene	Albaflavenone_biosynthetic_gene_cluster	100
Cluster 14	Siderophore	-	
Cluster 15	T1pks-Nrps	SW-163_biosynthetic_gene_cluster	39
Cluster 16	Bacteriocin	-	
Cluster 17	Terpene	-	
Cluster 18	Terpene	Hopene_biosynthetic_gene_cluster	92
Cluster 19	T1pks	Marineosin_biosynthetic_gene_cluster	81
Cluster 20	T1pks	Neoaureothin_biosynthetic_gene_cluster	81
Cluster 21	T1pks	ECO-02301_biosynthetic_gene_cluster	53
Cluster 22	Bacteriocin	Herboxidiene_biosynthetic_gene_cluster	3
Cluster 23	Nrps	Coelichelin_biosynthetic_gene_cluster	81
Cluster 24	Nrps	Actinomycin_biosynthetic_gene_cluster	14
Cluster 25	Nrps	Griseobactin_biosynthetic_gene_cluster	70
Cluster 26	Aryl polyene	Tetarimycin_biosynthetic_gene_cluster	5
Cluster 27	Lantipeptide	Toyocamycin_biosynthetic_gene_cluster	20
Cluster 28	Lantipeptide	Galbonolides_biosynthetic_gene_cluster	10
Cluster 29	Nrps	Mannopeptimycin_biosynthetic_gene_cluster	7

“-” corresponds to no similar known clusters.

## Data Availability

All the data in the article could be shared by the corresponding author upon request. They could be obtained in the article as follows: 1. The complete genomic sequence of* Streptomyces albireticuli* MDJK11 has been deposited in GenBank under accession number CP021744. 2. The complete genomic sequences of* S. alboflavus* MDJK44 have been deposited in GenBank under accession numbers CP021748, CP023976, and CP023977. 3. The potential secondary metabolite biosynthetic gene clusters were predicted out using antiSMASH v.4.1.0. 3.1 The corresponding data of strain MDJK44: https://antismash.secondarymetabolites.org/upload/bacteria-6a6b9832-50b7-40cd-b1a8-92dd7edcb6cf/index.html. 3.2 The corresponding data of strain MDJK44-pSJK1: https://antismash.secondarymetabolites.org/upload/bacteria-960e440c-9d7f-43aa-8de2-cb27150d46a5/index.html. 3.3 The corresponding data of strain MDJK44-pSJK2: https://antismash.secondarymetabolites.org/upload/bacteria-7615113d-891b-4bcf-b848-30741d4f8f78/index.html#. 3.4 The corresponding data of strain MDJK11: https://antismash.secondarymetabolites.org/upload/bacteria-59f2d283-9563-47da-a026-a9aaf0162895/index.html. 4. The genomic islands (GIs) were predicted by IslandViewer 4 and the data were presented in two Excel files.

## Supplementary Materials

Supplementary 1 Supplementary S1: the ANI analysis of* S. albireticuli* MDJK11 and* S. alboflavus* MDJK44.

Supplementary 2 Supplementary S2: qualitative analysis of siderophores on CAS-agar plates.
