# Supplementary material for: Screening and Whole-Genome Sequencing of Two Streptomyces Species from the Rhizosphere Soil of Peony Reveal Their Characteristics as Plant Growth-Promoting Rhizobacteria
Source: Biomed Res Int. 2018 Sep 5;2018:2419686. doi: 10.1155/2018/2419686 (PMC6145153; doi:10.1155/2018/2419686)
Supplement: Supplementary 1 — Supplementary S1: the ANI analysis of S. albireticuli MDJK11 and S. alboflavus MDJK44. [file 2419686.f1.docx]

SUPPLEMENTARY S1: The ANI analysis of two actinomycetes MDJK11 and MDJK44

|  | *Streptomyces_eurocidicus*_ATCC_27428 | ***Streptomyces_albireticuli*_strain_MDJK11** | *Streptomyces_albireticuli*_NRRL_B-1670^a^ | *Streptomyces_cinnamoneus*_ATCC_12686 | *Streptomyces_pactum*_ACT12 | *Streptomyces_olivaceus*_KLBMP_5084 | *Streptomyces_variegatus*_NRRL_B-16380 | ***Streptomyces_alboflavus*_strain_MDJK44** | *Streptomyces_alboflavus*_NRRL_B-2373 | *Streptomyces_fulvissimus*_DSM_40593 |
| --- | --- | --- | --- | --- | --- | --- | --- | --- | --- | --- |
| *Streptomyces_eurocidicus*_ATCC_27428 | --- | 92.87 | 92.62 | 87.23 | 85.05 | 84.91 | 84.82 | 85.01 | 85.01 | 85.18 |
| ***Streptomyces_albireticuli*_strain_MDJK11** | 92.86 | --- | **93.92** | 87.42 | 84.98 | 84.97 | 84.83 | 85.04 | 85.03 | 85.3 |
| *Streptomyces_albireticuli*_NRRL_B-1670^a^ | 92.63 | **93.97** | --- | 87.33 | 84.94 | 85.06 | 84.88 | 85.11 | 85.04 | 85.16 |
| *Streptomyces_cinnamoneus*_ATCC_12686 | 87.28 | 87.42 | 87.28 | --- | 85.51 | 85.44 | 85.46 | 85.42 | 85.39 | 85.63 |
| *Streptomyces_pactum*_ACT12 | 85.08 | 85 | 84.96 | 85.43 | --- | 89.27 | 86.67 | 85.36 | 85.37 | 84.85 |
| *Streptomyces_olivaceus*_KLBMP_5084 | 84.99 | 84.99 | 85.07 | 85.36 | 89.31 | --- | 86.42 | 85.45 | 85.39 | 84.8 |
| *Streptomyces_variegatus*_NRRL_B-16380 | 84.86 | 84.85 | 84.86 | 85.4 | 86.65 | 86.39 | --- | 85.23 | 85.19 | 84.9 |
| ***Streptomyces_alboflavus*_strain_MDJK44** | 85.12 | 85.09 | 85.12 | 85.42 | 85.38 | 85.46 | 85.27 | --- | **97.78** | 85.16 |
| *Streptomyces_alboflavus*_NRRL_B-2373 | 85.13 | 85.08 | 85.08 | 85.44 | 85.41 | 85.42 | 85.24 | **97.85** | --- | 85.13 |
| *Streptomyces_fulvissimus*_DSM_40593 | 85.28 | 85.28 | 85.14 | 85.62 | 84.84 | 84.79 | 84.89 | 85.14 | 85.09 | --- |
| ^a^The genome sequence of this strain is not the whole genome sequence. | | | | | | | | | | |
