# Supplementary material for: Screening and Whole-Genome Sequencing of Two Streptomyces Species from the Rhizosphere Soil of Peony Reveal Their Characteristics as Plant Growth-Promoting Rhizobacteria
Source: Biomed Res Int. 2018 Sep 5;2018:2419686. doi: 10.1155/2018/2419686 (PMC6145153; doi:10.1155/2018/2419686)
Supplement: Supplementary 2 — Supplementary S2: qualitative analysis of siderophores on CAS-agar plates. [file 2419686.f2.docx]

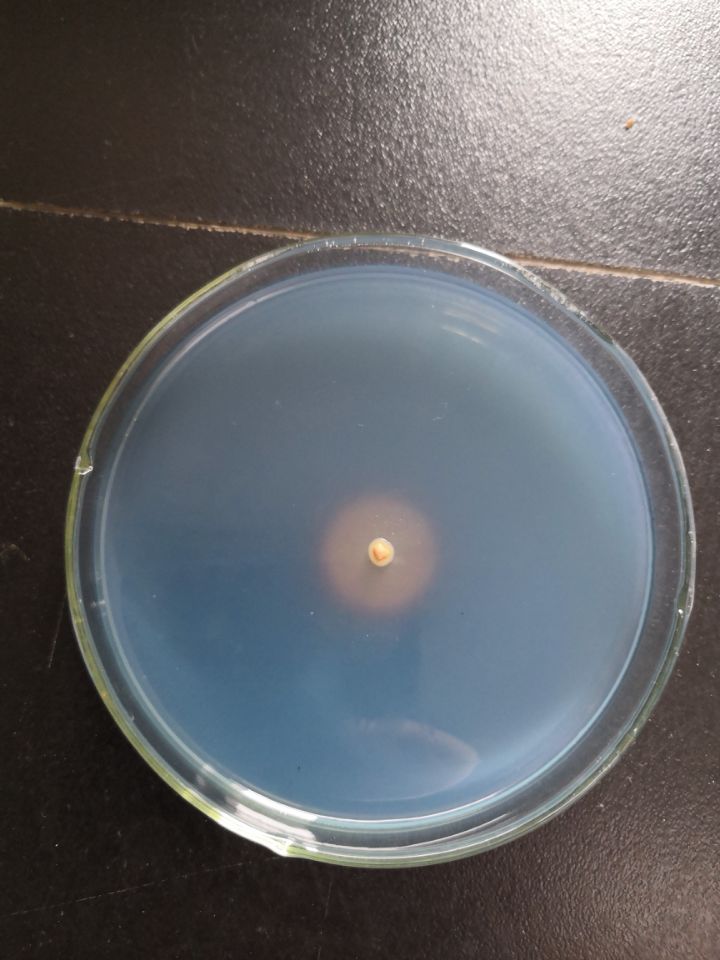

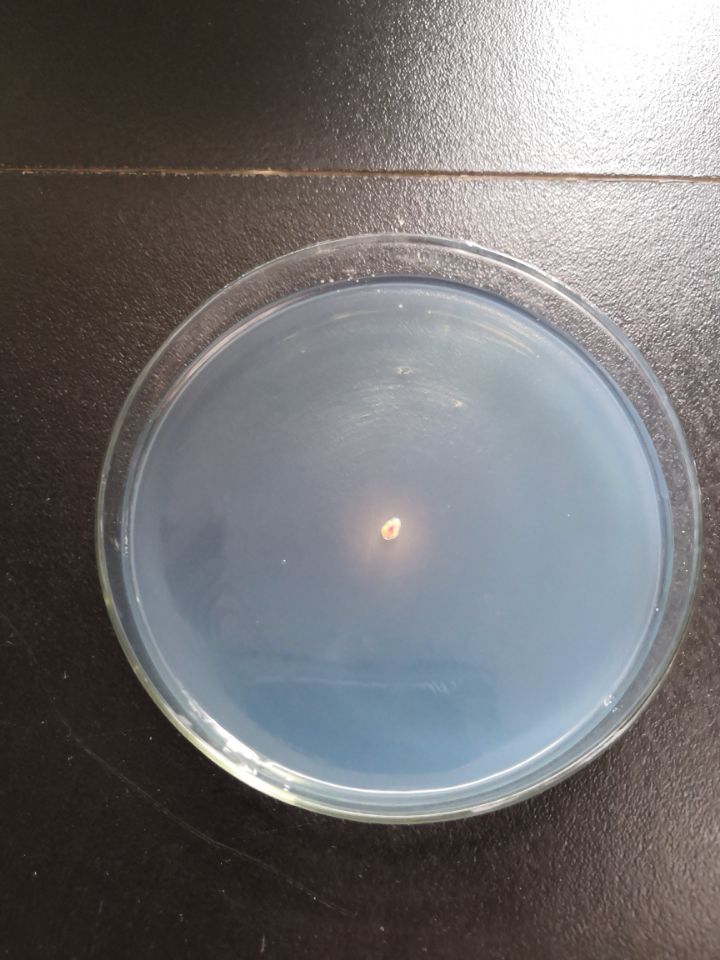


B

A

SUPPLEMENTARY S2: Qualitative analysis of siderophores on CAS-agar plates. Strains MDJK11 and MDJK44 were inoculated on the center of CAS-agar plates for cultivation at 28 °C for 3-5 days, respectively. The appearance of orange rings around the colonies suggested the production of siderophores. A) *S. albireticuli* MDJK11, B) *S.* *alboflavus* MDJK44.
